# Long-term cardiometabolic morbidity in young adults with classic 21-hydroxylase deficiency congenital adrenal hyperplasia

**DOI:** 10.1007/s12020-023-03330-w

**Published:** 2023-03-01

**Authors:** Beatrice Righi, Salma R. Ali, Jillian Bryce, Jeremy W. Tomlinson, Walter Bonfig, Federico Baronio, Eduardo C. Costa, Guilherme Guaragna-Filho, Guy T’Sjoen, Martine Cools, Renata Markosyan, Tania A. S. S. Bachega, Mirela C. Miranda, Violeta Iotova, Henrik Falhammar, Filippo Ceccato, Marianna R. Stancampiano, Gianni Russo, Eleni Daniel, Richard J. Auchus, Richard J. Ross, S. Faisal Ahmed

**Affiliations:** 1 Developmental Endocrinology Research Group, University of Glasgow, Royal Hospital For Sick Children, Glasgow, UK; 2 Department of Mother and Child, Azienda USL-IRCCS di Reggio Emilia, Reggio Emilia, Italy; 3 Office for Rare Conditions, Royal Hospital for Children & Queen Elizabeth University Hospital, Glasgow, UK; 4 Oxford Centre for Diabetes, Endocrinology & Metabolism, NIHR Oxford Biomedical Research Centre, Churchill Hospital, University of Oxford, Oxford, UK; 5 Department of Paediatrics, Technical University München, Munich, Germany; 6 Department of Paediatrics, Klinikum Wels-Grieskirchen, Wels, Austria; 7 Pediatric Unit, Department Hospital of Woman And Child, IRCSS S.Orsola-Malpighi University Hospital, Bologna, Italy; 8 Pediatric Surgery Service, Hospital de Clínicas de Porto Alegre, Porto Alegre, Brazil; 9 Department of Pediatrics, School of Medicine, Universidade Federal do Rio Grande do Sul (UFRGS), Porto Alegre, Brazil; 10 Department of Endocrinology - Center for Sexology and Gender, Ghent University Hospital, Ghent, Belgium; 11 Department of Internal Medicine and Paediatrics, Ghent University and Department of Pediatric Endocrinology, Ghent University Hospital, Ghent, Belgium; 12 Endocrinology, Yerevan State Medical University Endocrinology Clinic, Yerevan, Armenia; 13 Unidade de Endocrinologia do Desenvolvimento, Laboratório de Hormônios e Genética Molecular/LIM42, Disciplina de Endocrinologia, Hospital Das Clinicas, Faculdade De Medicina, Universidade de Sao Paulo, São Paulo, Brazil; 14 UMHAT Sveta Marina, Medical University of Varna, Varna, Bulgaria; 15 Department of Molecular Medicine and Surgery, Karolinska Institutet, Stockholm, Sweden; 16 Department of Endocrinology, Karolinska University Hospital, Stockholm, Sweden; 17 Endocrinology Unit, Department of Medicine DIMED, University-Hospital of Padua, Padua, Italy; 18 Department of Pediatrics, Endocrine Unit, IRCCS San Raffaele Scientific Institute, Endo-ERN Center for Rare Endocrine Conditions, Milan, Italy; 19 Department of Oncology and Metabolism, University of Sheffield, Sheffield, UK; 20 Division of Metabolism, Endocrinology and Diabetes, University of Michigan, Ann Arbor, MI USA

**Keywords:** 21-hydroxylase deficiency, Co-morbidities, Congenital adrenal hyperplasia, Outcome, Registry

## Abstract

**Purpose:**

To study the current practice for assessing comorbidity in adults with 21-hydroxylase CAH and to assess the prevalence of comorbidity in these adults.

**Methods:**

A structured questionnaire was sent to 46 expert centres managing adults with CAH. Information collected included current therapy and surveillance practice with a particular focus on osteoporosis/osteopaenia, hyperlipidaemia, type 2 diabetes/hyperinsulinaemia, hypertension, CV disease, obesity.

**Results:**

Of the 31 (67%) centres from 15 countries that completed the survey, 30 (97%) screened for hypertension by measuring blood pressure, 30 (97%) screened for obesity, 26 (84%) screened for abnormal glucose homoeostasis mainly by using Hb1Ac (73%), 25 (81%) screened for osteoporosis mainly by DXA (92%), 20 (65%) screened for hyperlipidaemia and 6 (19%) screened for additional CV disease. Of the 31 centres, 13 provided further information on the six co-morbidities in 244 patients with a median age of 33 yrs (range 19, 94). Of these, 126 (52%) were females and 174 (71%) received fludrocortisone in addition to glucocorticoids. Of the 244 adults, 73 (30%) were treated for at least one comorbidity and 15 (21%) for more than 2 co-morbidities. Of 73, the patients who were treated for osteoporosis/osteopaenia, hyperlipidaemia, type 2 diabetes/hyperinsulinaemia, hypertension, CV disease, obesity were 43 (59%), 17 (23%), 16 (22%), 10 (14%), 8 (11), 3 (4%) respectively.

**Conclusion:**

Cardiometabolic and bone morbidities are not uncommon in adults with CAH. There is a need to standardise the screening for these morbidities from early adulthood and to explore optimal therapy through routine collection of standardised data.

## Introduction

Congenital adrenal hyperplasia (CAH) is a group of autosomal recessive disorders characterised by a life-long deficiency of adrenal steroidogenic enzymes that leads to cortisol deficiency. In the commonest form of CAH due to 21-hydroxylase deficiency, the cortisol deficiency is accompanied by a variable extent of mineralocorticoid deficiency and androgen excess [1, 2]. Glucocorticoid replacement therapy in these patients aims to replace cortisol and prevent the ACTH-driven androgen excess. However, replacement therapy to normalise androgen levels can lead to excess glucocorticoid exposure with associated growth concerns, obesity, hypertension, osteoporosis and an adverse cardiometabolic profile [3–7]. Management guidelines for adults with CAH recommend an annual assessment of blood pressure and body mass index, in addition to an assessment of the adequacy of glucocorticoid and mineralocorticoid replacement [8]. However, the extent to which contemporary practice adheres or even exceeds these standards is unclear. This study was, therefore, performed to understand the current practice for assessing cardiometabolic and bone outcomes in adults with 21-hydroxylase CAH at expert centres. In addition to collecting information on practice, the study also aimed to assess the prevalence of cardiometabolic and bone comorbidity.

## Methods

**Fig. 1:**
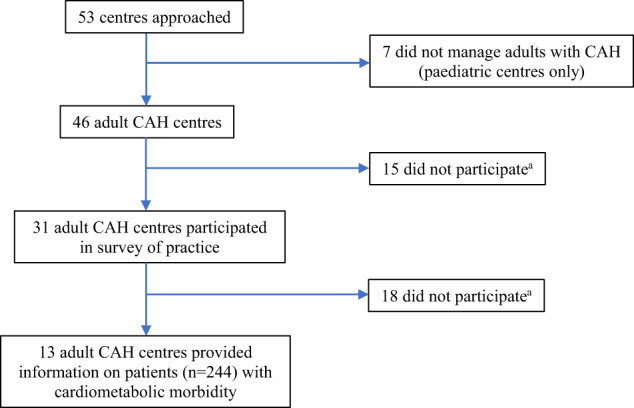
Consort diagram of the Study. ^a^Reasons for centres not participating included: Lack of time for data collection or completion (3), inadequate patient information (4), no response (26)

Between January 2020 and August 2020, centres that had previously been members of the Congenital Adrenal Hyperplasia Adult Study Executive (CaHASE) Consortium in the UK [9], centres that were members of the European Reference Network on Rare Endocrine Conditions (Endo-ERN) [10] and centres that contributed data to the I-CAH Registry [11] were invited to participate in a survey of current practice. For I-CAH, centres were identified by searching the Registry for patients who were aged 18 years and over with 21-hydroxylase CAH. The I-CAH Registry (https://home.i-cah.org) is an international database collecting pseudonymized information on patients with CAH and is approved by National Research Ethic Service in the United Kingdom as a research database of information that is collected as part of routine clinical care [11]. The survey was sent to 40 centres that had patients in the I-CAH Registry that were 18 yrs old and over at the time of the survey. Of these, 33 were actively involved in the management of adults with CAH. In addition, the survey was sent to 8 centres that were previously within the CaHASE consortium and 5 additional centres from Endo-ERN that were not within I-CAH or CaHASE and were endorsed as Endo-ERN reference centres that managed adults with CAH. Of these 46 centres that were actively managing adults with CAH, 31 (67%) from 15 countries completed the survey. These 31 centres included 18 from I-CAH, 8 from CaHASE and 5 from Endo-ERN (Fig. 1). The survey included questions on the practice of screening for cardiometabolic and bone co-morbidities including the methods used and the frequency of assessment (Online Resource). In the second part of the study, centres were asked to provide routinely collected clinical information on all patients with classic 21-hydroxylase CAH who had received therapy for obesity, type 2 diabetes mellitus (T2DM), hyperlipidaemia, hypertension, cardiovascular (CV) disease or osteoporosis and of the 31 centres, 13 (42%) from 8 countries provided data on a total of 244 adults. The data were described using Microsoft Excel (2019).

## Results

### Survey of current practice

Of the 31 centres, 30 (97%) screened for hypertension by measuring blood pressure, 30 (97%) screened for obesity by using body mass index (BMI) (90%) and weight (83%), 26 (84%) screened for abnormal glucose homoeostasis by mainly using Hb1Ac (73%) or fasting plasma glucose (50%), 25 (81%) screened for osteoporosis mainly by dual-energy X-ray absorptiometry (DXA) (92%), 20 (65%) screened for hyperlipidaemia using fasting lipids and 6 (19%) routinely screened patients for additional CV disease (Table 1).

**Table 1:** Centres screening for cardiometabolic comorbidity in adult patients with classic congenital adrenal hyperplasia and screening methods used

Comorbidity	Screening for cardiometabolic comorbidity, *n* (%)			Frequency of screening, *n* (%)				
	Centres	Methods	Centres performing each method	Every visit	6 monthly	1 to 2 yearly	Other^a^	Not specified
Hypertension	30 (97)	Systolic and diastolic BP	30 (100)	1 (3)	3 (10)	9 (30)	1 (3)	16 (54)
		24-hour BP measurement	6 (20)	–	–	–	2 (33)	4 (64)
		Nocturnal BP measurement	–	–	–	–	–	–
Obesity	30 (97)	BMI	27 (90)	2 (7)	2 (7)	8 (30)	–	15 (56)
		Weight	25 (83)	3 (12)	1 (4)	5 (20)	–	16 (64)
		Waist circumference	9 (30)	–	1 (11)	3 (33)	–	5 (56)
		Hip circumference	4 (13)	–	–	1 (25)	–	3 (75)
Abnormal glucose homoeostasis	26 (84)	HbA1c	19 (73)	1 (5)	–	8 (42)	3 (16)	7 (37)
		Fasting plasma glucose	13 (50)	–	–	5 (38)	2 (16)	6 (46)
		Random plasma glucose	12 (46)	–	–	7 (58)	–	5 (42)
		Fasting plasma insulin	8 (31)	–	–	3 (38)	1 (12)	4 (50)
		HOMA index	8 (31)	1 (13)	–	2 (25)	2 (25)	3 (37)
		2-hour blood glucose	7 (27)	–	–	1 (14)	3 (43)	3 (43)
		Insulin resistance index	–	–	–	–	–	–
Hyperlipidaemia	20 (65)	Fasting HDL cholesterol	13 (65)	–	–	5 (38)	–	8 (62)
		Fasting triglycerides	13 (65)	–	–	5 (38)	–	8 (62)
		Fasting total cholesterol	12 (60)	–	–	5 (42)	–	7 (58)
		Fasting LDL cholesterol	11 (55)	–	–	4 (36)	–	7 (64)
		Others^b^	6 (30)	–	–	–	–	6 (100)
Cardiovascular disease	6 (19)	Cardiac USS	2 (33)	–	–	–	1 (50)	1 (50)
		Stress test	–	–	–	–	–	–
		Cardiac MRI	–	–	–	–	–	–
		Others^b^	5 (83)	–	–	–	–	5 (100)
Osteoporosis	25 (81)	DEXA	23 (92)		≤5 yearly (4, 17%), >5 yearly (8, 35%)			11 (48)
		Others^b^	4 (16)	–	–	–	–	4 (100)

*BP* blood pressure, *BMI* body mass index, *Hb1Ac* haemoglobin 1Ac, *HOMA* homoeostatic model assessment of insulin resistance, *HDL* high density lipoprotein, *LDL* low density lipoprotein, *USS* ultrasound scan, *MRI* magnetic resonance imaging, *DXA* dual X-ray absorptiometry

^a^Other included only if problems identified (e.g. if random blood pressure (BP) reading elevated (*n* = 3), random hyperglycaemia (*n* = 7)) or screening every 2 years or less frequently (*n* = 5)

^b^Other screening methods: For hyperlipidaemia included non-fasting lipid profile (*n* = 4), lipid profile intermittently from age 40 (*n* = 1), unknown (*n* = 1); for cardiovascular disease included intima media thickness (IMT) USS evaluation (*n* = 2), history and clinical evaluation (*n* = 2), Electrocardiogram (*n* = 1); for osteoporosis included lumbar X-ray (*n* = 1), Q-risk fracture (*n* = 1), bone ultrasonography (*n* = 1), fracture score after 50’s (*n* = 1)

### Description of cases

The median age of the 244 cases that were included in the study of cardiometabolic morbidity was 33 years (range 19, 94). Overall, 126 (52%) were females and 118 (48%) males; 158 (65%) had been clinically reported to have salt wasting CAH and the remaining 35% had simple virilizing CAH. However, of the 244, 174 (71%) were reported to have taken fludrocortisone. For glucocorticoid (GC) replacement, 99 (41%) patients were receiving prednisolone, 89 (36%) hydrocortisone and 20 (8%) dexamethasone; 31 (13%) patients were receiving a combination of glucocorticoids: cortisone acetate-dexamethasone (*n* = 16, 7%), hydrocortisone-prednisolone (*n* = 4, 2%), cortisone acetate-dexamethasone-prednisolone (*n* = 4, 2%), prednisolone-dexamethasone (*n* = 3, 1%), hydrocortisone-dexamethasone (*n* = 2, 1%), cortisone acetate-prednisolone (*n* = 1, 0.4%). In a further 3 cases, the type of GC medication was unclear and in two cases GC replacement was only reserved for stress related events.

### Cardiometabolic and bone morbidity

Of the 244 cases, 73 (30%) patients were on therapy for at least one of the six cardiometabolic and bone co-morbidities that were assessed (Table 2). Of these 73, 9 (12%) were on treatment for 2 co-morbidities, 3 (4%) were on treatment for 3 co-morbidities and 3 (4%) were on medication for 4 co-morbidities. The commonest therapy that 43 (59%) of the 73 patients were receiving was for osteoporosis or osteopenia; 33 (77%) of these 43 patients were males and 28 (65%) were treated with prednisolone. The age at start of bone-protective therapy was 39 (range 18–63). The co-morbidities that had the youngest age at start of therapy were those related to glucose homoeostasis. Among 16 patients requiring therapy for abnormal glucose homoeostasis, females and males were equally represented and 11 had salt wasting CAH. Of the 10 cases who were on antihypertensive therapy, 5 had salt wasting CAH and were on therapy with fludrocortisone. All obese patients with CAH had salt wasting form and were treated with fludrocortisone. A wide range of drugs were used for each cardiometabolic condition, especially for cardiovascular disease, hyperlipidaemia and hypertension. In two cases of hypertension, combination therapy of two or three drugs was reported (Table 2).

**Table 2:** Baseline characteristics of the 73 patients with classic congenital adrenal hyperplasia (30%) on therapy against different cardiometabolic and bone co-morbidities

Cardiometabolic comorbidity	Sex *n* (%)		CAH type *n* (%)		FC therapy *n* (%)	Patients on drug therapy, *n* (%)				Drug therapy used *n* (%)			
	Female	Male	SW	SV		HC	Pred	Dex	Combination	Total, *n* (%)	Start age (years)	Duration (years)	Type of Therapy
Osteoporosis	7 (32)	15 (68)	13 (59)	9 (41)	14 (64)	5 (23)	15 (68)	1 (4)	1 (4)	22 (9)	39 (18, 63)	4 (0, 17)	Vitamin D, 9 (41) Calcium + Vitamin D, 9 (41) Alendronate + Calcium + Vit D, 2 (9) Calcium, 1 (5) Risedronate, 1 (5)
Osteopaenia	3 (14)	18 (86)	12 (57)	9 (43)	17 (81)	7 (33)	13 (62)	0	1 (5)	21 (9)	32 (18, 55)	6 (0, 12)	Calcium + Vitamin D, 13 (62) Vitamin D, 6 (29) Calcium, 2 (9)
Hyperlipidaemia	6 (35)	11 (65)	10 (59)	7 (41)	11 (65)	5 (29)	6 (35)	3 (18)	3 (18)	17 (7)	55 (19, 79)	3 (0, 10)	Atorvastatin, 6 (35) Simvastatin, 5 (29) Pravastatin, 3 (18) Fenofibrate, 1 (6) Rosuvastatin/Ezetimibe, 1 (6) Not known, 1 (6)
T2 Diab Mellitus	2 (40)	3 (60)	2 (40)	3 (60)	2 (40)	4 (80)	1 (20)	0	0	5 (2)	26 (21, 78)	0 (0, 9)	Insulin, 3 (60) Metformin, 2 (40)
Hyperinsulinaemia	5 (45)	6 (55)	9 (82)	2 (18)	8 (73)	3 (27)	1 (9)	0	7 (64)	11 (5)	25 (14, 55)	3 (1, 8)	Metformin, 10 (91) Not known, 1 (9)
Hypertension	6 (60)	4 (40)	5 (50)	5 (50)	5 (50)	8 (80)	1 (10)	1 (10)	0	10 (4)	55 (39, 72)	2 (0, 14)	ACE inhibitors, 3 (30) Calcium channel blockers, 2 (20) Loop diuretics, 1 (10) Beta blockers, 1 (10) ≥2 drugs, 2 (20)^a^ Not known, 1 (10)
Cardiovascular disease	3 (38)	5 (62)	4 (50)	4 (50)	4 (50)	2 (25)	6 (75)		0	8 (3)	65 (55, 72)	7 (0, 10)	Anti-platelets, 3 (38) ≥2 drugs, 2 (25)^b^ Anticoagulants, 2 (25) Surgery for interatrial communication, 1 (12)
Obesity	2 (67)	1 (33)	3 (100)	0	3 (100)	0	2 (67)	0	1 (33)	3 (1)	24 (19, 28)	2 (1, 3)	Orlistat, 1 (33) Sibutramine, 1 (33) Bariatric surgery, 1 (33)

*CAH* Congenital Adrenal Hyperplasia, *N* number of patients for each characteristic, *FC* fludrocortisone, *F* females, *M* males, *SW* salt wasting forms, *SV* simply virilizing forms, *HC* hydrocortisone, *CA* cortisone acetate, *Pred* prednisolone, *Dex* dexamethasone

^a^Combination includes angiotensin receptor antagonist and beta blockers (*n* = 1) and beta-blocker plus calcium channel blocker plus ace inhibitor (*n* = 1)

^b^Combination includes antiplatelets plus beta-blocker plus ace inhibitor plus nitroglycerin (*n* = 1), antiplatelet plus beta-blocker plus long-acting nitroglycerin (*n* = 1)

## Discussion

This is the first multicentre study that has assessed the current practice of monitoring cardiometabolic and bone conditions in a large sample of adults with classic 21-hydroxylase deficiency CAH. Previous studies have reported the prevalence of cardiometabolic abnormalities in CAH, but the actual use of medications has rarely been reported [3]. The findings were particularly remarkable given the relatively young age of the reported cohort. Given the variable definition of these co-morbidities, we employed the actual use of a medication as a more pragmatic marker for a clinically important comorbidity. This strategy has allowed us to understand the range of co-morbidities and the therapies that were used.

Bone-protective therapy was the commonest group of drugs that was used in this cohort and in the majority consisted simply of calcium and/or vitamin D supplements. Studies of DXA-assessed bone mineral density (BMD) have shown inconsistent findings in CAH, probably reflecting the multiple determinants of bone health that play an important role in the maintenance of bone health in people with CAH. However, a recent meta-analysis reported slightly reduced BMD in adults with CAH [12] and an increasing number of studies [13–15] have reported a higher prevalence of fractures. Moreover, a recent national register-based study found that fracture prevalence was increased in both sexes in those with classic CAH compared to matched controls [15]. Whilst the Endocrine Society guidelines recommend bone health monitoring in only those who are at high risk of adverse bone health [8], others recommend a more pragmatic approach with a routine DXA scan at 2–5 yr intervals in all adults who are on glucocorticoid treatment for CAH and especially when the doses are supraphysiologic [16]. The current study showed that around 80% of centres had taken this latter approach to bone health monitoring. Whilst there may also be a rationale for considering bone-protective therapy in those with low BMD, those who are on high dose glucocorticoids or have co-existing hypogonadism, there is a need for further study of larger cohorts to understand the long-term benefit of bone-protective therapy.

The prevalence of obesity in adulthood seems to vary from one population to another in CAH, with a higher prevalence of obesity in patients in some countries, such as the UK (41%) [17] and Sweden where the risk of obesity was almost 11 times higher in patients than in healthy controls [18]. In other countries such as the US [5] and France [19], the prevalence of obesity has been reported to be similar to the background population (33% and 22% respectively). Almost all the centres monitored obesity by assessing weight and BMI, and some centres also routinely measured waist or hip circumferences. Over 80% of centres also screened for abnormal glucose homoeostasis and in the majority, screening was performed by measuring HbA1c while some centres measured fasting glucose and insulin. In adults with CAH, abnormal glucose homoeostasis has indeed been previously reported [5, 17] but in a recent meta-analysis, HOMA-IR was the only marker of insulin resistance that was reported to be marginally high [6]. However, two large registry-based studies demonstrated higher prevalence of diabetes [18] and gestational diabetes in patients with CAH [20]. Rarely have these previous studies reported on concomitant medication for these co-morbidities. Of the 199 adults included in the CaHASE cohort, 5% were on antihypertensives and 1% were on oral hypoglycaemic agents [21], compared to 7% and 5%, respectively in the current study. It is possible that some of the differences between the two studies in the use of the oral hypoglycaemic agents may be due to the relative proportion of cases in the two cohorts who were on dexamethasone, as the proportion of patients on dexamethasone were lower in the CaHASE cohort compared to the current cohort [3], but this will require more detailed studies. In the current study, it was notable that there were 5 cases of T2DM, 3 of whom were on insulin, the use of which has not been widely described in patients with CAH. Given that the average age for starting pharmacologic treatment for T2DM is around 65 years in Europe [22], the age at starting therapy either with metformin or insulin was much earlier in the current study cohort. The use of metformin has been shown to improve biochemical and clinical outcomes in non-classic congenital adrenal hyperplasia (NC-CAH) [23], but its use in classic CAH has rarely been described [24] and requires further study.

Several studies have also examined the lipid profile in adults with CAH, and some have reported normal results [25–28] while others have reported hypercholesterolaemia [4, 17]. The actual use of lipid-lowering agents in CAH was 3% in the current study and similar to the report by Han et al. [21] and also similar to the use of lipid-lowering agents in the general adult population [29]. Statins (particularly atorvastatin and simvastatin) have been studied particularly in NC-CAH females with hyperlipidaemia, showing an improvement not only in lipids but also in androgen levels [30, 31] and these were the commonest group of drugs that were used in the current cohort too.

While individual studies have reported normal or high diastolic and systolic blood pressure, the meta-analysis by Tamhane et al. had identified hypertension as a significant finding [6] and in addition to weight, the Endocrine Society guidelines have recommended the regular monitoring of blood pressure [8]. The current study shows that blood pressure measurement was routinely performed. In the general population, about 12% of adults between the ages of 20 and 40 years may suffer from hypertension and in a large proportion of these individuals, the hypertension may be secondary to a predisposing condition [32]; across the age span in adults this increases to 20–30% [33]. The prevalence of antihypertensive therapy in the current study was about 7% and similar to a previous report [26]. The median age at start of therapy in the current study was over 50 yrs suggesting that hypertension that required therapy was not particularly evident in early adulthood. It was also interesting to note the wide range of antihypertensive therapies that were used in this group of patients, including beta blockers, angiotensin converting enzyme (ACE) inhibitors, calcium channel blockers, angiotensin receptor antagonist and loop diuretics. Given the wide variety of medications that were being used as antihypertensives sometimes in concurrent use of fludrocortisone, the relative merit of these regimens in CAH should be compared over the longer term with continued and routine collection of data.

In addition to obesity, abnormal glucose homoeostasis, dyslipidaemia and hypertension, studies in adults with CAH have revealed a wide range of cardiovascular concerns including increased carotid intima thickness [6], diastolic dysfunction [34] increased thromboembolic risk [18] as well as dysrhythmia [18]. However, the clinical significance of these findings, their relationship to the clinical characteristics of the cases and long-term outcome need to be systematically studied. The uncertainty of whether routine cardiovascular assessment should be performed or not was reflected in the current study where only a fifth of centres performed any routine assessment, and even when this was performed, a variety of methods were used to do so. We believe there is a need to develop a consensus amongst expert centres on what should be considered a core set of cardiovascular assessments that should be performed routinely. The prevalence of cardiovascular disease that required therapy was only 3%, but given that a relatively small percentage of patients were routinely monitored, this proportion may not represent the extent of pathology.

The current study was performed as an overview of the range of cardiometabolic co-morbidities that are encountered in adults with CAH. It is likely that our findings are an underestimate, as we have only reported the instances when these patients required therapy [35], without agreeing among participating centres on a clear cut-off when to start treatment. It is possible that some patients may have sub-clinical findings, especially in those situations where routine monitoring was not performed. The current study provides a simple snapshot of the health status of young adults with CAH. However, to calculate more accurate lifetime risks of these important co-morbidities in such a rare condition, there is a need to continue collecting standardised information in registries such as the I-CAH Registry, complemented with more detailed studies where relevant. This effort would also be dependent on achieving greater consensus on the core set of measures that should be assessed routinely as part of clinical practice. The study has also highlighted that several expert centres follow a more rigorous regimen of monitoring adults with CAH than what has been generally recommended, raising the need for a review of the current guidance in this field. Lastly, there are other co-morbidities such as pyschiatric disorders or their therapy that have been previously been described to be commoner in adults with CAH and which were not studied here [36–38].

In conclusion, current practice for assessing cardiometabolic and bone outcomes in adults with CAH exceeds the recommended guidance and is variable. The therapeutic management of the co-morbidities that were encountered in CAH in this study is more variable for some conditions such as hypertension than others. Given the rarity of CAH and the occurrence of cardiometabolic and bone co-morbidities, it is imperative that centres engage in collective efforts that facilitate the understanding of the long-term risk factors for these morbidities and develop evidence-based pathways for their management.

## Supplementary Information

Supplementary Table
