# Supplementary material for: Long-term cardiometabolic morbidity in young adults with classic 21-hydroxylase deficiency congenital adrenal hyperplasia
Source: Endocrine. 2023 Mar 1;80(3):630–8. doi: 10.1007/s12020-023-03330-w (PMC10199864; doi:10.1007/s12020-023-03330-w)
Supplement: Supplementary file 1 — Supplementary Table [file 12020_2023_3330_MOESM1_ESM.pdf]

Supplementary Table List of questions and possible answers asked in the survey of current practice for assessing the presence of adverse cardiometabolic comorbidities

**Question**	**Responses available**	**Response type**
Clinician details		Free text
Clinician name		
Centre name		
e-mail		
In the case of a patient who is aged 18 years or over:		
Do you screen patients with CAH for abnormal glucose homeostasis?	Yes/No	Yes/No
What methods do you use to screen for abnormal glucose homestasis and how often?	Random plasma glucose	Select all responses that apply and specify how often the method is used in free text
	Fasting plasma glucose	
	Fasting plasma insulin	
	HOMA index	
	Insulin Resistance Index (IRI)	
	2 h blood glucose (OGTT)	
	Hb1Ac	
	Other (please specify)	
Do you screen patients with CAH for hyperlipidaemia?	Yes/No	Yes/No
What methods do you use to screen for hyperlipidaemia and how often?	Fasting total cholesterol	Select all responses that apply and specify how often the method is used in free text
	Fasting LDL cholesterol	
	Fasting HDL cholesterol	
	Fasting triglycerides	
	Other (please specify)	
Do you screen patients with CAH for hypertension?	Yes/No	Yes/No
What methods do you use to screen for hypertension and how often?	Systolic and diastolic BP	Select all responses that apply and specify how often the method is used in free text
	24 h BP measurement	
	Nocturnal BP measurement	
	Other (please specify)	

Do you screen patients with CAH for cardiovascular disease?	Yes/No	Yes/No
What methods do you use to screen for cardiovascular disease and how often?	Stress test	Select all responses that apply and specify how often the method is used in free text
	Cardiac USS	
	Cardiac MRI	
	Other (please specify)	
Do you screen patients with CAH for obesity?	Yes/No	Yes/No
What methods do you use to screen for obesity and how often?	Weight	Select all responses that apply and specify how often the method is used in free text
	BMI	
	Waist circumference	
	Hip circumference	
	Other (please specify)	
Do you screen patients with CAH for osteoporosis?	Yes/No	Yes/No
What methods do you use to screen for osteoporosis and how often?	Dual X-ray absorptiometry (DEXA)	Select all responses that apply and specify how often the method is used in free text
	Other (please specify)	

CAH: Congenital Adrenal Hyperplasia; HOMA: Homeostasis Model Assessment of insulin resistance; OGTT: oral glucose tolerance test; LDL: low density lipoprotein; HDL: high density lipoprotein; BP: blood pressure; USS: ultrasound scan; MRI: magnetic resonance imaging; BMI: body mass index; I-CAH: international congenital adrenal hyperplasia registry
